# Health Sciences Online: an extraordinary opportunity for the democratization of health sciences knowledge

**DOI:** 10.1186/1743-8454-7-S1-S53

**Published:** 2010-12-15

**Authors:** Erica Frank

**Affiliations:** 1 School of Population and Public Health, University of British Columbia, 5804 Fairview Avenue, Vancouver, BC, V6T 1E3, Canada

## Background

According to the World Health Organization, health care requires more innovative, trans-disciplinary and less-expensive training methods to increase local education and service opportunities. We are addressing this issue through Health Sciences Online (HSO) - a virtual learning centre for comprehensive health professional education. HSO is a portal that provides access to a collection of top-quality courses and references in medicine, public health, nursing, dentistry and other health sciences disciplines. These materials are donated, hosted and maintained by distinguished content partners so anyone, anywhere in the world can access a free, current, world-class education through the portal.

## Materials and methods

HSO includes more than 50,000 learning objects from already-existing reliable resource collections, and we are still growing. Material regarding hydrocephalus and spina bifida is available, provided by medical specialty societies, accredited continuing education organizations, governments and universities (including the Centre for Genetics Education, the Canadian Paediatric Society, and the University of Pittsburgh School of Medicine).

## Results

As our next phase, we're beginning work with colleagues all over the world in creating what we hope to be the largest, most accessible, and one of the best health sciences universities -- all done with distance HSO-based didactics, local hands-on mentoring, and peer-to-peer distance feedback. We plan to train many thousands of trainees at a time, particularly in developing countries, with the students remaining in their home environments (and thereby building capacity, instead of encouraging brain drain). Examples of certificates currently under development are in 1) Exercise and Health, in partnership with CDC, the American College of Sports Medicine, the Fundacion Santa Fe Bogota Active Living Program, and the Pedagogical University of Colombia, 2) Perinatal Care, in collaboration with WHO, and piloting in Africa and South East Asia, 3) Emerging Infectious Diseases, with WHO, and piloting in a rural community in Panama, 4) Dermatology for Primary Care Providers, in collaboration with NATO and Armenia’s Yerevan State Medical University and 5) Addiction Medicine for Medical Students and Residents, in collaboration with the Betty Ford Institute and the Annenberg Physician Training Program in Addiction Medicine.

## Conclusions

HSO is an extraordinary resource for medical professionals around the world, and we hope to utilize this conference to teach others about its origins, uses and goals.

